# Role of Cigarette Smoking on Serum Angiotensin-Converting Enzyme and Its Association With Inflammation and Lipid Peroxidation

**DOI:** 10.7759/cureus.27857

**Published:** 2022-08-10

**Authors:** Dinesh Nath, Meera Shivasekar

**Affiliations:** 1 Biochemistry, Sri Ramaswamy Memorial (SRM) Medical College Hospital, Chennai, IND; 2 Biochemistry, Sri Ramaswamy Memorial (SRM) Medical College Hospital and Research Center, Sri Ramaswamy Memorial Institute of Science and Technology (SRMIST), Chennai, IND

**Keywords:** mda, cigarette smoking, inflammation, coronary heart disease, angiotensin-converting enzyme

## Abstract

Introduction

Cigarette smoking promotes angiotensin-converting enzyme (ACE) production and causes a substantial change in inflammation and oxidative stress, resulting in an increase in antioxidant activity and lipid peroxidation.

Objective

The study's goal is to determine the role of cigarette smoking on serum ACE and its relation with inflammatory markers and lipid peroxidation.

Methods

The cross-sectional study consists of three groups. The study participants are all men between the age group of 20 to 55 years. Group 1 includes 120 healthy controls as nonsmokers, Group 2 consists of 120 active smokers with coronary heart disease (CHD) and Group 3 includes 120 active smokers with diabetic CHD patients attending the SRM Medical College Hospital in Tamil Nadu for cardiology and medical Outpatient. Measurements of serum ACE, oxidized low-density lipoprotein (oxLDL), high-sensitivity C-reactive protein (hsCRP), and matrix metalloprotease-9 (MMP-9) were performed using the ELISA method (enzyme-linked immunosorbent assay). Using a spectrophotometric approach, the total antioxidant capacity and lipid peroxidation, particularly Malondialdehyde (MDA), were assessed.

Results

The mean serum ACE (92.35±10.28), oxLDL (48.59±8.56), hs-CRP (5.87±1.62), MMP-9 (89.20±30.19), and MDA (1.146±0.198) levels were significantly (p-value <0.0001) higher in smokers with CHD and diabetes (group 3) when compared to group 1 and group 2. On the other hand, the total antioxidant capacity (0.413±0.097) of smokers of group 3 was found to be (p<0.0001) significantly lower than those of group 1 and group 2. The study also demonstrated a significant correlation between ACE with MDA, ox-LDL, total antioxidant capacity, hs-CRP, MMP-9, smoking load, and smoking intensity in smokers.

Conclusion

The study concludes a substantial correlation exists in smokers owing to ACE modification, which results in inflammation and lipid peroxidation activation. This is strongly associated with an increased risk of major cardiovascular events.

## Introduction

Cardiovascular disease (CVD) is the major cause of death in developing countries. Smoking, hypertension, diabetes mellitus (DM), and hypercholesterolemia are all substantial risk factors [1]. There is emerging evidence that nicotine use and smoking status have an impact on the severity and mortality of cardiovascular disease [2]. However, the precise mechanisms through which these factors induce CVDs remain unknown. The renin-angiotensin-aldosterone system (RAAS), a peptide-based system, has traditionally been viewed as a complicated linear humoral system for maintaining cardiovascular disease risk homeostasis, and it appears to play a role in the development of cardiovascular disease [3]. Studies have suggested that in systemic circulation angiotensin-converting enzyme (ACE) is produced in the pulmonary endothelial cell caused by cigarette smoking (smoking). It is also hypothesized that ACE activity may increase the changes in cardiovascular events associated with smoking [4]. Systemic ACE is found on the endothelium's surface and is involved in the conversion of circulating angiotensin I to angiotensin II [5]. The vascular action of angiotensin II is primarily mediated by the AT1 receptor subtype, a 7-transmembrane G-protein-coupled receptor that not only promotes vascular smooth muscle vasoconstriction but also activates endothelin-1, a strong vasoconstrictor [5, 6]. By promoting endothelial synthesis of plasminogen activator inhibitor-1, angiotensin II improves monocyte adherence to endothelial cells and lowers local fibrinolytic activity in vitro [7]. It is significant to note that angiotensin II has been shown to affect vascular structural integrity by modifying the formation and breakdown of extracellular matrix and by encouraging lipid oxidation [8]. There is mounting evidence that oxidized LDL (ox-LDL) contributes to atherogenesis and endothelial damage. In atherosclerotic artery walls, oxidized-LDL (ox-LDL) and its receptors have been identified [9]. As a chemotactic factor for monocytes and a cytotoxic substance, ox-LDL causes an influx of inflammatory cells and the production of free radicals produced from oxygen [10]. The involvement of the RAAS system in inflammation has been more well-known in recent years. Several studies have shown that the ACE/Ang II/AT1R and the ACE2/Ang-(1-7)/Mas receptor axis are two significant RAAS counterregulatory mechanisms that balance pro-inflammatory activities [11]. Ang II is now known to be a pro-inflammatory molecule [12]. Recent research suggests that Ang II activates AT1R, and has a crucial function in modulating the production of cytokines such as hsCRP, MMP-9, IL-2, NO, and tumor necrosis factor- (TNF-) in monocytes and macrophages [13]. So, the aim of the study is to find out the role of cigarette smoking on serum angiotensin-converting enzyme activity in smokers and its relation to inflammation and lipid peroxidation.

## Materials and methods

Study design and subjects

The present cross-sectional study was conducted at SRM Medical College Hospital and Research Center, SRMIST, between November 2020 and September 2021. Based on the prevalence rate the study group was divided into three groups. Group 1 includes 120 healthy controls as nonsmokers, group 2 includes 120 young active smokers with coronary heart disease (CHD) and group 3 includes 120 young active smokers with diabetic CHD subjects who were attending the SRM Medical College Hospital in Tamil Nadu for cardiology and medicine outpatient. The Human Research Ethical Committee of SRM Medical College Hospital and Research Center, SRMIST, sanctioned the study in accordance with the ethical standards of the Indian Council of Medical Research (IEC No: 1763). Initially, all participants are given a basic questionnaire to identify the patients who are qualified to participate in the study based on the inclusion/exclusion criteria. The study's participants are all men between the ages of 20 and 55. Patients who smoked regularly and had been diagnosed with CHD met the inclusion criteria. The diagnosis of CHD was based on typical chest pain, ECG changes suggestive of ischemia, abnormal lab value (CPK, CK-MB, and troponin I), abnormal echocardiographic changes, and abnormal coronary angiography with more than 50% stenosis of one of the major coronary artery. Patients with cardiomyopathy, chronic illnesses such as liver failure, cancer patients, heart failure, cerebrovascular accidents, severe systemic illness, and systemic inflammatory disease were excluded from the study.

Ascertainment of smoking exposure

Self-reporting was used to determine smoking status. Smokers were defined as people who smoked conventional cigarettes frequently >5 cigarettes per day for at least the previous 12 months considered in the study [14].

Protocol and measure

The study participants were instructed to arrive at the hospital between 8:00 and 9:00 a.m. following a minimum of 12 hours of overnight fasting and overnight rest with no physical activity prior to laboratory measures. Serum samples were obtained by centrifuging blood samples at 2000 g for 10 minutes at 4 degrees Celsius and storing them at -20 degrees Celsius until analysis. All the analyses were performed at the central laboratory of SRM Medical College Hospital and Research Center. Age, gender, educational history, and other health and medical histories are determined via self-reporting. Standard equipment and technique were used to measure anthropometric characteristics such as weight and heights. After 2 minutes of rest, resting blood pressure was measured three times at one-minute intervals in the sat posture; the average of the second and third measurements was used for analysis. Hypertension was described as having a systolic blood pressure of more than 140 mmHg and diastolic blood pressure of more than 90 mmHg. A previous medical diagnosis of diabetes mellitus or meeting diagnostic criteria for diagnosis based on fasting plasma glucose levels of ≥126 mg/dl, 2-hour plasma glucose levels obtained as part of a 75-gm oral glucose tolerance test of ≥200, or the glycated hemoglobin test (HbA1c) of ≥6.5 percent were used to define diabetes mellitus. Fasting glucose level, total cholesterol level, triglyceride level, high-density lipoprotein cholesterol (HDL-C) level, low-density lipoprotein level, and very-low-density lipoprotein level were all measured enzymatically in the AU480 automated analyzer (Beckman Coulter, Brea, CA, USA).

Analysis of ACE

The serum ACE concentration was determined using a sandwich enzyme-linked immunosorbent assay (ELISA) (Abbkine Scientific Co., Ltd., Wuhan, China).

hsCRP and MMP-9 assay

The serum hsCRP and MMP-9 concentrations were determined using a sandwich enzyme-linked immunosorbent assay (ELISA) (hsCRP; BioCheck, Inc., South San Francisco, CA, USA, and MMP-9 from Abbkine Scientific Co., Ltd., Wuhan, China).

Ox-LDL and MDA assay

According to the manufacturing protocol, the serum ox-LDL (normal range ≤30 ug/L) concentration was determined using a sandwich enzyme-linked immunosorbent assay (ELISA) (Abbkine Scientific Co., Ltd., Wuhan, China). Serum malondialdehyde (MDA) analysis is dependent on the reaction with thiobarbituric acid (TBA) to create thiobarbituric acid reactive substances (TBARS) that may be measured colorimetrically (532 nm).

Total antioxidant capacity assay

According to the manufacturing process, a widely available colorimetric kit (Abbkine Scientific Co., Ltd., Wuhan, China) was used to measure the total antioxidant capacity of serum using the FRAS (ferric reducing ability of serum) technique.

Statistical analysis

For the statistical analysis, IBM Corp.'s Statistical Package for the Social Sciences (SPSS) version 22 was employed (IBM Corp., Armonk, NY, USA). The mean standard deviation is used to express quantitative quantities. To compare mean values for continuous variables, descriptive analysis was done using one-way ANOVA. A multivariable regression analysis was carried out using ACE as a dependent variable and taking the independent variables to assess the contribution of ACE to the prediction of CHD of each parameter. Accordingly, a linear regression analysis was performed using ACE as the dependent variable to see if the association of ACE versus all biochemical markers was altered by smoking status. A "p-value" of less than 0.05 was considered statistically significant.

## Results

Baseline and biochemical characteristics of the study groups

The demographic and baseline characteristics of controls (nonsmokers), smokers with CHD, and smokers with diabetic CHD are shown in Table 1 and Table 2. This study included 360 participants who were divided into three groups. Group 1 consists of 120 healthy non-smokers, group 2 of 120 young active smokers with CHD, and group 3 of 120 young active smokers with diabetic CHD. Weight, BMI, WC, HC, W/H ratio, BP, number of cigarettes smoked per day, and duration of smoking all showed a significant difference. Fasting blood glucose, lipid profile, and lipid ratio were statistically more significant in smokers in groups 2 and 3 when compared to group 1 (controls).

**Table 1:** Anthropometric measurements of smokers with CHD and normal controls. p<0.05 is statistically significant. One-way ANOVA calculation. BMI: body mass index, WC: waist circumference, HC: hip circumference, W/H ratio: waist/hip ratio, CHD: coronary heart disease.

Parameter	Control group 1 (n=120)	Smokers with CHD group 2 (n=120)	Smokers with CHD and diabetes group 3 (n=120)	p-value
Age (years)	33.40±10.76	38.81±8.55	42.76±6.87	<0.0001
Height (cm)	170.4±3.67	170.95±5.39	171.69±5.14	0.424
Weight (kg)	68.62±7.16	73.75±6.85	75.58±8.03	<0.0001
BMI (kg/m^2^)	23.47±3.11	25.64±2.71	25.71±2.19	<0.0001
WC (cm)	87.16±5.43	91.84±5.73	92.71±4.91	<0.0001
HC (cm)	99.75±4.36	102.18±3.56	101.61±4.69	0.013
W/H ratio	0.87±0.14	0.90±0.12	0.91±0.07	<0.0001
Systolic BP (mmHg)	116.6±4.9	127.2±7.7	134.8±7.2	<0.0001
Diastolic BP (mmHg)	83.5±4.4	85.1±4.1	88.8±7.2	<0.0001
Number of Smoking/day	0	8.29±3.31	9.8±3.9	<0.0001
Duration of Smoking(years)	0	15.96±8.14	18.15±6.23	<0.0001

**Table 2:** Biochemical parameters of smokers with CHD and normal controls (non-smokers). p<0.05 is statistically significant. One-way ANOVA calculation. FBG: fasting blood glucose, TC: total cholesterol, TGL: triacylglycerides, HDL: high-density lipoprotein, LDL: low-density lipoprotein, VLDL: very low-density lipoprotein, RLP-C: remnant lipoprotein cholesterol, ACE: Angiotensin-converting enzyme, oxLDL: oxidized low-density lipoprotein, MDA: malondialdehyde, TAC: total antioxidant capacity, hs-CRP: C-reactive protein, MMP-9: Matrix metalloproteinase-9.

Parameter	Control group 1 (n=120)	Smokers with CHD group 2 (n=120)	Smokers with CHD and diabetes group 3 (n=120)	p-value
FBG (mg/dl)	100.7±9.15	102.7±10.18	216.81±61.77	<0.0001
TC (mg/dl)	155.55±21.9	216.45±35.19	229.11±44.61	<0.0001
TGL (mg/dl)	100.21±41	177.29±69.51	214.30±79.23	<0.0001
HDL (mg/dl)	45.81±7.92	40.28±5.93	38.95±4.88	<0.0001
LDL (mg/dl)	105.9±11.91	158.45±21.22	166.25±23.43	<0.0001
VLDL (mg/dl)	20.81±8.27	34.12±16.87	42.28±24.41	<0.0001
TC/HDL	3.59±0.59	5.45±0.82	5.99±3.07	<0.0001
LDL/HDL	2.6±0.41	3.86±0.62	4.31±0.84	<0.0001
RLP-C [TC − (HDL-C + LDL-C)]	10.52±6.27	20.98±14.23	44.15±21.27	<0.0001
Non HDL-C	112.677±21.88	176.64±25.42	189.86±56.71	<0.0001
Non HDL-C/HDL	2.76±0.81	4.18±0.49	5.11±2.28	<0.0001
Non HDL-C/TC	0.7±0.04	0.81±0.07	0.82±0.03	<0.0001
ACE (U/L)	46.94±9.49	77.84±9.31	93.85±10.71	<0.0001
OX-LDL (ug/L)	25.72±3.11	39.54±4.68	48.19±9.51	<0.0001
MDA (mmol/L)	0.561±0.037	0.786±0.320	1.141±0.238	<0.0001
TAC (mmol/L)	1.171±0.24	0.591±0.141	0.431±0.094	<0.0001
hsCRP (mg/L)	0.756±0.331	3.49±1.29	5.77±1.48	<0.0001
MMP-9 (ng/ml)	27.41±9.49	66.17±23.16	88.30±31.51	<0.0001

The study shows that smokers in groups 2 and 3 had considerably higher serum ACE levels. When compared to controls (Group 1) smokers with diabetic CHD subjects had the highest level of serum ACE.

When compared to CHD subjects without diabetes, inflammatory markers (hsCRP, MMP-9) gradually increased in smokers with diabetes (“p-value” 0.0001), indicating that smokers with CHD who have a complication of diabetes significantly increase the systemic inflammation.

Table 2 shows the concentrations of serum oxLDL levels when compared to controls. Group 3 cases exhibit a marked elevation of oxLDL levels as compared to group 2. The serum MDA levels were slightly higher in group 2 and group 3 when compared to controls. When compared to controls, smokers with diabetic CHD patients had the highest amount of serum MDA, which was approximately two times higher (“p-value” <0.0001).

When compared to controls, the study demonstrates a gradual decline in serum total antioxidant capacity in smokers with CHD, followed by diabetic CHD subjects (“p-value” <0.0001). The findings indicated the systemic imbalances between oxidants and antioxidants in smokers.

**Figure 1:**
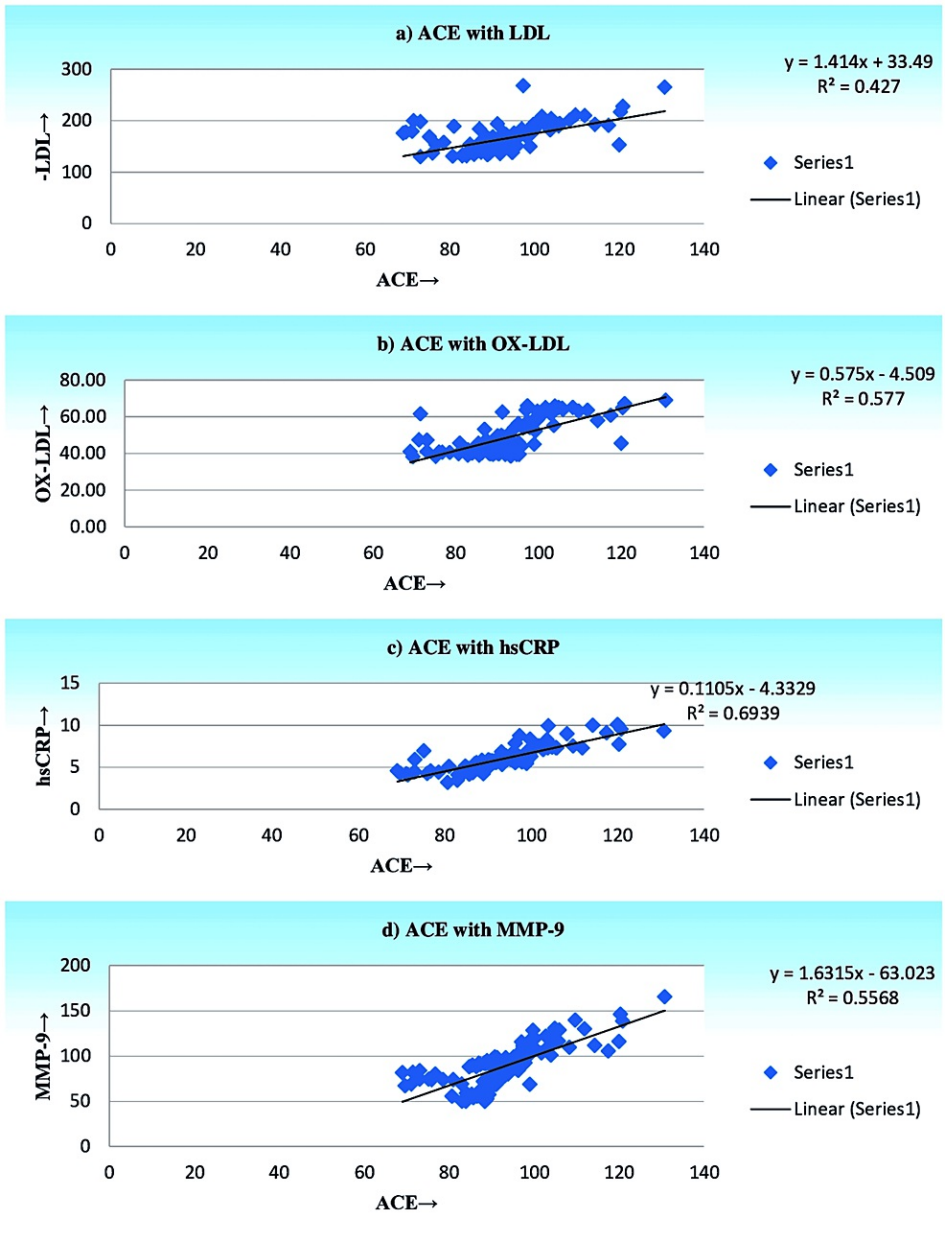
Linear regression analysis of ACE with LDL, OX-LDL, hs-CRP, and MMP-9 in smokers with diabetic CHD subjects. ACE: angiotensin-converting enzyme, LDL: low-density lipoprotein, OX-LDL: oxidized low-density lipoprotein, hs-CRP: high-sensitivity C-reactive protein, MMP-9: matrix metalloprotease-9, CHD: coronary heart disease.

**Figure 2:**
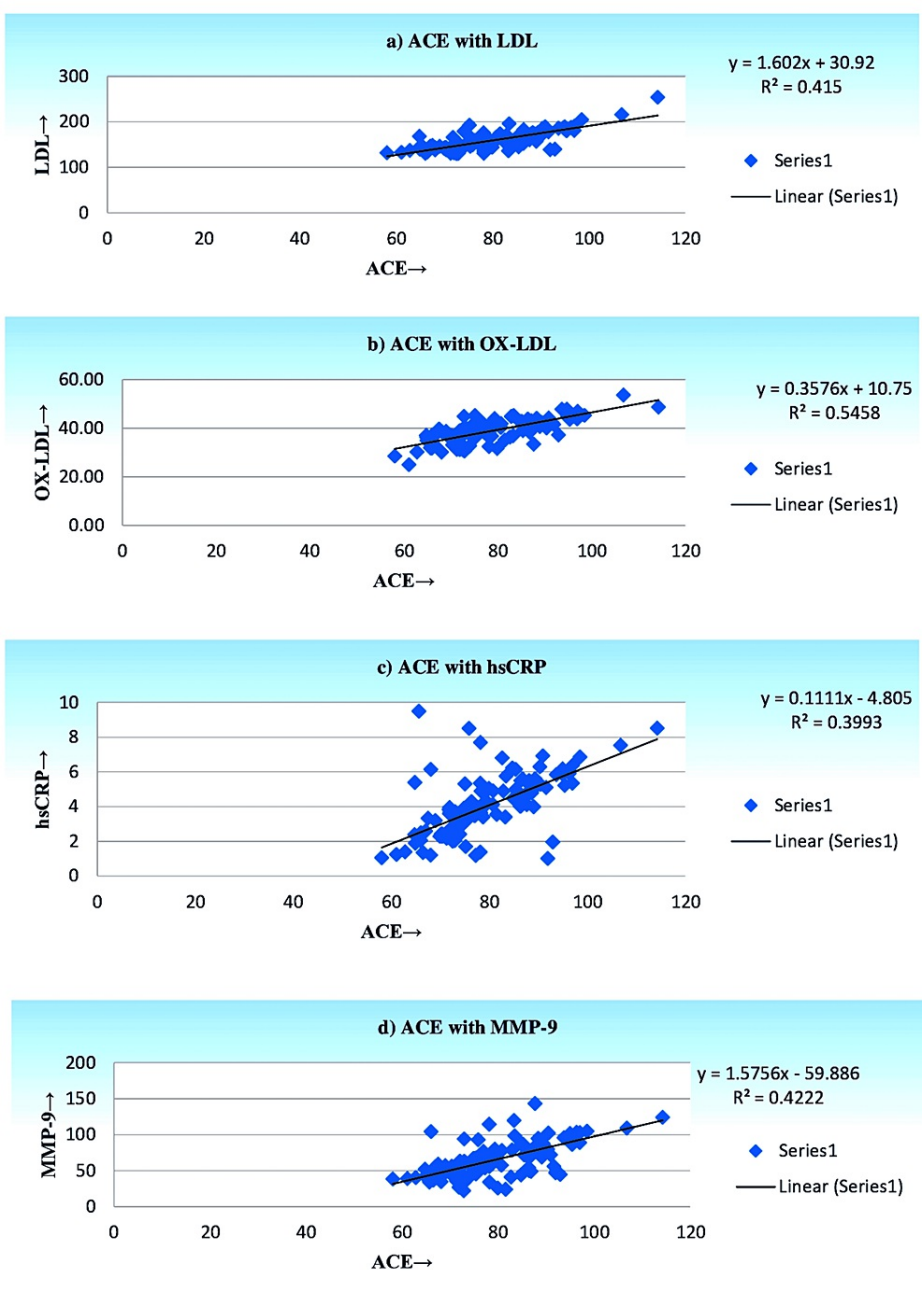
Linear regression analysis of ACE with LDL, OX-LDL, hs-CRP, and MMP-9 in smokers with CHD subjects. ACE: angiotensin-converting enzyme, LDL: low-density lipoprotein, OX-LDL: oxidized low-density lipoprotein, hs-CRP: high-sensitivity C-reactive protein, MMP-9: matrix metalloprotease-9, CHD: coronary heart disease.

The linear regression analysis of ACE with LDL, OX-LDL, hsCRP, and MMP-9 in smokers with CHD and diabetic CHD individuals is shown in Figure 1 and Figure 2. A significant positive correlation was observed between the LDL, OX-LDL, hsCRP, and MMP-9 with ACE scores in smokers with CHD subjects. Moreover, LDL, OX-LDL, hsCRP, and MMP-9 were all significantly and positively correlated with ACE scores in smokers with diabetic CHD subjects. Diabetic CHD subjects exhibit a strong significant association with ACE scores when compared to CHD subjects.

Multivariable analyses

In Table 3 the multivariate regression analysis was performed to determine independent predictors for CHD in smokers taking the ACE level as the dependent variable showed that HDL (p=0.044), LDL (p=0.008), MDA (p=0.013), OX-LDL (p=0.001), total antioxidant capacity (p=0.016), hs-CRP (p=0.011), MMP-9 (p=0.015), smoking intensity (p=0.020) and smoking burden (p=0.003) were significantly associated with higher ACE level.

**Table 3:** Multivariate regression analysis of the CHD risk with all biochemical parameters of smokers with CHD subjects. p<0.05 is statistically significant. Cl: Confidence interval. The multivariate regression analyses were adjusted for all biochemical parameters taking ACE as a dependent variable. CHD: coronary heart disease, MDA: malondialdehyde, ox-LDL: oxidized low-density lipoprotein, hs-CRP: high-sensitivity C-reactive protein, MMP-9: matrix metalloprotease-9, TC: total cholesterol, TAG: triacylglycerol, HDL: high-density lipoprotein, LDL: low-density lipoprotein, VLDL: very low-density lipoprotein.

Variables	Standardized β	p-value	(95%Cl)
MDA	.154	.014	(-2.240 – 12.914)
OX-LDL	.273	.001	(.231 – .910)
TOTAL ANTIOXIDANT	-.153	.016	(-6.523 – 4.935)
hs-CRP	.183	.012	(-.329 – 1.320)
MMP-9	.151	.015	(-.022 – .161)
TC	.122	.145	(-.076 – .168)
TAG	.119	.148	(-.006 – .042)
HDL	-.110	.044	(-.401 – -.009)
LDL	.332	.008	(.0423 – .272)
VLDL	.043	.573	(-.160 – .076)
SMOKING INTENSITY	.162	.020	(.090 – 1.062)
SMOKING BURDEN	.235	.003	(.153 – .443)

In Table 4, we also performed the multivariate regression analysis to determine independent predictors for CHD and diabetes in smokers taking the ACE level as the dependent variable showed that triacylglycerol (TAG) (p=0.014), HDL (p=0.013), LDL (p<0.001), MDA (p<0.0001), ox-LDL (p<0.001), total antioxidant capacity (p=0.002), hs-CRP (p=0.001), MMP-9 (p=0.001), smoking intensity (p=0.002) and smoking burden (p=0.003) were significantly associated with higher ACE level. The study indicated that the higher ACE concentration had a significantly higher risk of CHD and diabetes in smokers after adjustment for the established risk factors.

**Table 4:** Multivariate regression analysis of the CHD risk with all biochemical parameters of smokers with diabetic CHD subjects. The multivariate regression analyses were adjusted for all biochemical parameters taking ACE as a dependent variable. Cl: Confidence interval, CHD: coronary heart disease, MDA: malondialdehyde, ox-LDL: oxidized low-density lipoprotein, hs-CRP: high-sensitivity C-reactive protein, MMP-9: matrix metalloproteinase-9, TC: total cholesterol, TAG: triacylglycerol, HDL: high-density lipoprotein, LDL: low-density lipoprotein; VLDL: very low-density lipoprotein.

Variables	Standardized β	p-value	(95%Cl)
MDA	.305	<0.001	(-6.434 – 6.771)
OX-LDL	.589	<0.001	(.464 – .968)
T. ANTIOXIDANT	-.262	.002	(-7.235 – 19.979)
hs-CRP	.298	.001	(2.858 – .766)
MMP-9	.275	.001	(-.031 – .187)
TC	.134	.133	(-.007 – .056)
TAG	.153	.014	(-.041 – .119)
HDL	-.156	.013	(-.486 – -.064)
LDL	.462	<0.001	(.119 – .244)
VLDL	.021	.902	(.126 – .159)
SMOKING INTENSITY	.265	.002	(.127 – .273)
SMOKING BURDEN	.234	.003	(.151 – .432)

## Discussion

Smoking unquestionably has a negative impact on the cardiovascular system. In order to better understand how smoking affects ACE and to shed light on how smoking may affect the cardiovascular system, we looked at the relationship between smoking and ACE. Numerous researches have revealed that nicotine or smoking increases systolic and diastolic blood pressure. The result of our findings also suggested that smoking increases systolic and systolic blood pressure [15], and these effects were accompanied by increasing ACE concentration by disrupting the renin-angiotensin-aldosterone system. Multiple epidemiologic studies have revealed that serum ACE levels are abnormally increased in patients with cardiovascular disease and hypertension and are connected to coronary artery stenosis. The renin-angiotensin-aldosterone system plays a significant role in the progression of cardiovascular disease [16]. In this study, smokers with CHD had significantly higher blood ACE levels than nonsmokers (control) (p<0.0001), suggesting that ACE may alter angiotensinogen 2 levels, which can lead to major consequences such as coronary heart disease. Previously, it was observed that patients who survived a myocardial infarction (MI) had greater plasma ACE levels [17]. They reported that patients under the age of 55 had higher levels of ACE. Thus, the findings of our and prior investigations show that smoking interacts with the renin-angiotensin system in some way [18].

Cardiovascular complications are common among diabetics, although the mechanisms behind these complications remain unknown. Plasma ACE levels were shown to be higher in people with diabetes (p<0.05). The most obvious possibility is that ACE and ACE2, by regulating the levels of Ang II and/or Ang-(1-7) in pancreatic islets, are involved in the control of insulin secretion to the extent that blood flow is influenced by local levels of angiotensin peptides. This finding of our study is consistent with the previous research [19, 20] and might be explained by the higher frequency of CHD in the diabetic population.

The different study clearly shows that smokers with CHD had a decreased level of antioxidant status and a greater level of lipid peroxidation than nonsmokers (controls). These investigations proved that smoking cigarettes might have harmful consequences on its own. In our study, we choose to evaluate serum antioxidant capacity, two lipid peroxidation indicators (MDA and oxLDL) as well as the two inflammatory markers, hsCRP and MMP-9. The data of the present study indicate that young smokers with CHD have a lower serum antioxidant capacity when compared to controls. The current study found that the amounts of MDA released from erythrocytes in smoker CHD patients were considerably higher than in healthy controls which is consistent with the previous study done by Bhat et al. [21, 22].

Our research has also focused on the oxidative alteration of lipids, particularly LDL, and the possible significance of ox-LDL in the development of atherosclerosis [23]. Oxidized LDL is much more toxic than native LDL, causing foam cells to form and the formation of early lesions [24]. Studies have demonstrated that oxLDL enhances platelet adhesion, breaks DNA strands, and induces cell death, all of which contribute to the progression of atherosclerotic disease [25] and have been identified as an important marker in relation to oxidative stress research.

This current study shows a significant positive association between cardiovascular risk factors especially smoking with (hsCRP) and MMP-9. Moreover, different studies show that the elevated levels of MMP-9 were positively linked with smoking status and inflammatory markers (including hsCRP, and IL-6) [26].

Serum MMP-9 levels were significantly higher in smokers with diabetic CHD individuals compared to CHD without diabetes and the control group (p<0.0001). This might be due to abnormal extracellular matrix metalloproteinase synthesis in diabetic CHD patients [27].

Our investigation also shows a substantial significant positive relationship between serum ACE and an increase in smoking load and smoking intensity in both the groups and was consistent with a previous study performed in Boston, USA [28, 29]. It is indicated that serum ACE levels in young smokers are significantly associated with the severity of smoking status. The levels can be used as a sensitive marker for evaluating the extent of coronary heart disease.

Our study has some limitations. Our analysis shows ancestry accounts for variability in plasma ACE levels in our population, so we cannot generalize the results to other populations. The study design and methods used were not suitable for distinguishing genetic from environmental effects or addressing clinical implications of the observed differences in ACE levels between groups. Additionally, we were not able to include female smokers because smoking disclosed to women is socially unacceptable in the southern part of India. To understand the communication between ACE, inflammation, and lipid peroxidation in smokers much more work remains to be done. Addressing these issues is crucial for a better understanding of ACE and for developing therapeutic strategies to target the consequences of CHD.

## Conclusions

In conclusion, our results illustrated an independent connection between ACE serum levels and smokers with CHD and diabetes. The study shows that smoking increases the synthesis of angiotensin-converting enzyme (ACE) and significantly alters the inflammatory status. The study also reveals that ACE is elevated by aberrant lipid levels, most likely through ox-LDL synthesis. Smoking, on the other hand, causes the release of reactive oxygen species by activating ACE, with a concomitant increase in lipid peroxidation, and reducing the antioxidant deface system which increases the risk of coronary heart disease to a large extent.
